# Determination of the crystalline size of hexagonal La_1−*x*_Sr_*x*_MnO_3_ (*x* = 0.3) nanoparticles from X-ray diffraction – a comparative study

**DOI:** 10.1039/d3ra04018f

**Published:** 2023-08-22

**Authors:** Do Hung Manh, Tran Thi Ngoc Nha, Le Thi Hong Phong, Pham Hong Nam, Tran Dang Thanh, Pham Thanh Phong

**Affiliations:** a Institute of Materials Science, Vietnam Academy of Science and Technology 18-Hoang Quoc Viet Hanoi City Vietnam thanhxraylab@yahoo.com; b Graduate University of Science and Technology, Vietnam Academy of Science and Technology 18-Hoang Quoc Viet Hanoi City Vietnam; c Laboratory of Magnetism and Magnetic Materials, Science and Technology Advanced Institute, Van Lang University Ho Chi Minh City Vietnam phamthanhphong@vlu.edu.vn; d Faculty of Applied Technology, School of Technology, Van Lang University Ho Chi Minh City Vietnam

## Abstract

The electronic, magnetic, optical and elastic properties of nanomaterials are governed partially by the crystallite size and crystal defects. Here, the crystalline size of hexagonal La_1−*x*_Sr_*x*_MnO_3_ (*x* = 0.3) nanoparticles was determined using various methods. Single-phase La_0.7_Sr_0.3_MnO_3_ nanopowders were produced after 10 h of milling in a commercial high-energy SPEX 8000D shaker mill, and then they were heated at 700 °C and 800 °C to study the effect of calcined temperature on the crystallization of nanoparticles. The modified Scherrer, Williamson–Hall, size–strain, and Halder–Wagner methods were used to determine the crystallite sizes and the elastic properties, such as intrinsic strain, stress, and energy density, from the X-ray diffraction peak broadening analysis. The obtained results were then compared with one another. The difference in crystallite sizes calculated from the different methods was due to the different techniques.

## Abstract 2

The electronic, magnetic, optical and elastic properties of nanomaterials are governed partially by the crystallite size and crystal defects.

## Graphical abstract

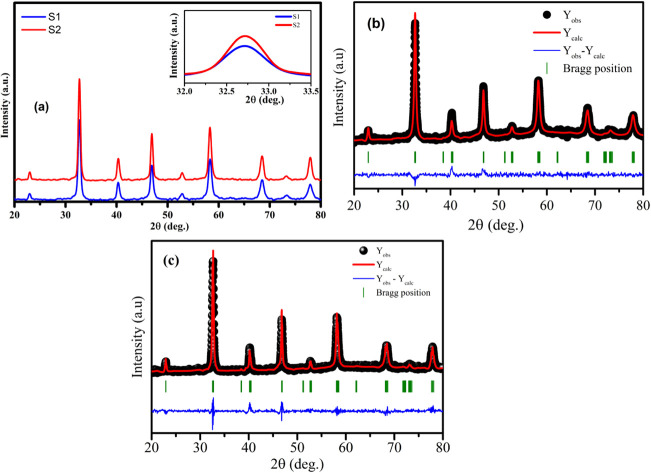

## 1. Introduction

La_1−*x*_Sr_*x*_MnO_3_ materials with perovskite structure of the type ABO_3_ have been the subject of much research interest in solid-state physics for a long time, because they exhibit many interesting physical effects, especially the strong correlation between their magnetic and transport properties.^1,2^ These properties are closely related to the ionic size effect, which depends on the applied chemical pressure, *i.e.*, whether or not the ion is substituted in site A or B, resulting in modification of the bond distance and thus the bandwidth (or electron hopping interaction).^3^ The kinetic energy of conduction electrons is controlled by the doping level, which not only drives the metal insulator transition but also governs the competing magnetic interaction.^4^ Among all perovskite manganites, the La_0.7_Sr_0.3_MnO_3_ (*x* = 0.3) compound is one of the most promising materials due to the high magnetic moment at room temperature and large Curie temperatures (*T*_C_ ∼ 370 K).^5^ Thus, it exhibits significant colossal magneto-resistance (CMR) and magnetocaloric effects, and holds great potential for applications in magnetic refrigeration (MR) and magnetic recording technology.^6^ However, materials with *T*_C_ values close to room temperature are important technological requirements.^7^ Researchers showed that partial substitution of the ions at site A or B by other metal ions and reduction in their particle size to a critical value (usually nanometers) could make the *T*_C_ values reach close to room temperature, which is beneficial for MR technology.^8–11^

Considering the crystal structure of the La_1−*x*_Sr_*x*_MnO_3_ system, most reports indicated that the rhombohedral structure could be observed in the range of 0.2 ≤ *x* ≤ 0.5.^12,13^ However, Bindu^3^ found that the hexagonal structure exists in the samples *x* = 0.2, 0.3, and 0.4, similar to the observation in the La_0.7_Sr_0.3_MnO_3_ nanopowders fabricated by high-energy ball milling and then calcined at 700 °C, 800 °C, and 900 °C.^11^ Through appropriate fabrication methods, the La_0.7_Sr_0.3_MnO_3_ manganite could clearly be either a rhombohedral lattice structure or hexagonal and orthorhombic ones. In nanocrystals, size confinement gives rise to large intrinsic strain, and this important elastic characteristic could be tuned by different synthesis parameters, such as pH, calcined temperature, and concentration of pre-chemicals.^15^ Usually, the crystallinity of a sample could be evaluated by analyzing different peaks of X-ray diffraction (XRD).^14^ For nanocrystals, a broadening of the XRD peaks could be observed due to the finite size effect and the existence of an intrinsic strain derived from the size confinement. Hence, a physical peak broadening mainly consists of two parts: one is related to size-dependent broadening, and the other is due to the contribution of strain-induced broadening.^15^ The most ordinary sources of the lattice strain are point defects, contact or sinter stress, grain boundary junction, dislocation density, and stacking faults.^16–18^ Therefore, the size of nanocrystals; the value of the intrinsic strain; and other elastic properties, such as stress and energy density, which are related to strain, could be determined indirectly through XRD peak broadening analysis. Many methods could be used for this analysis, including the Williamson–Hall (W–H), Warren–Averbach, and Balza methods. Among them, the W–H method utilizes the full-width half-maximum (FWHM) of the diffraction peak. Therefore, this method is very easy to apply and the acceptable one for determining different elastic properties, including strain, and calculating the average crystalline size.

Until now, limited attention has been paid to the microstructural parameters and crystal defects of nanomanganites. Most works only focused on studies related to crystallite size and grain size calculation, without investigating crystal defects and the internal stresses, uniform strain, lattice stress, and deformation energy density of nanomanganites. These structural parameters greatly influence the physical properties of the material. Thus, studying these parameters could not only provide important additional information about the crystal structure of manganites but also show the effect of stress on their crystallite size.

The present work is a continuation of a previous study on the same nanoparticle samples, in which the effect of calcined temperature on the magnetic and AC magnetic heating characteristics of La_0.7_Sr_0.3_MnO_3_ nanopowders have been reported.^11^ The present work aimed to study the crystallographic properties, microstructural parameters, and crystal defects of these nanoparticles. A comparative study of these microstructural properties of prepared La_0.7_Sr_0.3_MnO_3_ nanopowders based on XRD peak broadening was conducted using different models, such as the modified Scherrer, Williamson–Hall, size–strain plot (SSP), and Halder–Wagner methods. Subsequently, morphological analysis was performed *via* Field emission scanning electron microscopy (FESEM), and the average size obtained from this method was compared to the obtained results from XRD analysis.

## 2. Experimental procedures

La_0.7_Sr_0.3_MnO_3_ nanopowders were prepared using reactive milling combined with thermal processing methods as reported in the authors' previous work.^11^ After 10 h of milling in ambient atmosphere, the resulting powder was calcined at 700 °C and 800 °C for 4 h in air. The samples obtained were denoted as S1 for sample calcined at 700 °C and S2 for sample calcined at 800 °C. The phase purity, homogeneity, and crystal structure were characterized by XRD using a Bruker D8 Advance X-ray diffractometer with CuKα radiation (*λ* = 1.5406 Å) and an accelerating voltage of 40 kV. The data were recorded at room temperature from 20° to 80° for 2*θ* at a scanning speed of 2° min^−1^ and a step size of 0.02°. The XRD patterns were studied by the commercial X'pert Highscore Plus of the PANalytical program for Rietveld refinement analysis. The surface morphology of the samples was observed using scanning electron microphotographs under Field emission scanning electron microscopes (Hitachi S-4800).

## 3. Results and discussion

**Fig. 1:**
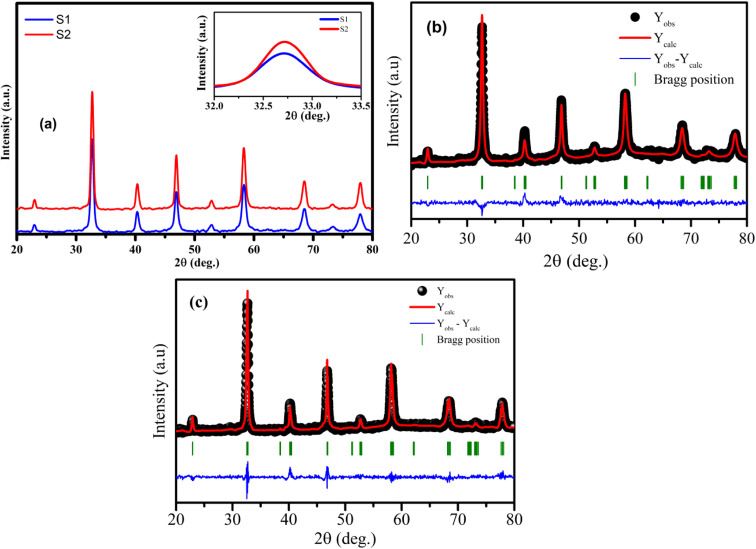
(a) XRD patterns of S1 and S2 manganites. The inset shows the diffraction angle of the intense peak. (b) and (c) Rietveld refinement for the S1 and S2 samples.

The analysis of phase purity and structural properties of samples at room temperature were done using X-ray diffraction (XRD) measurements. Fig. 1(a) displays the XRD patterns of both studied samples La_1−*x*_Sr_*x*_MnO_3_ (*x* = 0.3) sintered at 700 °C (S1) and 800 °C (S2). All the samples are single phasic crystallizing in perovskite phase, with no trace of any impurity phases. The experimental data were analyzed using the X'Pert HighScore Plus software, which confirmed that all the samples were crystallized in the hexagonal symmetry and the *R*3̄*C* space group. The inset of Fig. 1(a) shows the intensity of the main diffraction peaks of La_0.7_Sr_0.3_MnO_3_ nanopowders. The intensity of the main peak clearly increased as the annealed temperature increased from 700 °C to 800 °C, indicating the improvement of *x* = 0.3 crystallinity at higher annealed temperature. Besides, the full-width half-maximum (FWHM) of the main *x* = 0.3 peaks decreased as the annealed temperature increased. As shown in Fig. 1(b) and (c), the Rietveld analysis was applied in samples S1 and S2. The experimental Bragg reflections were consistent with their calculated positions. The *a*- and *c*-axis lattice parameters increased slightly with the sintering temperature, which is in very well agreement with the authors' previous report.^11^ From this diffraction data, the crystal sizes and related structural parameters of the samples were calculated, and the obtained results are discussed in the following section.

### 3.1. The modified Scherrer method

The crystalline size of nanomaterials is usually calculated using Scherrer's formula, which is based on the broadening of the diffraction peaks from the size effect and intrinsic strain effect. Given that the diffraction peak broadening includes physical and instrumental broadenings,^19,20^ it must be corrected using the following equation:

1 *β*_d_^2^ = *β*_m_^2^ + *β*_i_^2^,

where *β*_m_ is the measured broadening, *β*_i_ is the instrumental broadening, and *β*_d_ is the corrected broadening responsible for crystalline size. Here, crystalline silicon was used as the reference material for calibration error from instrumental broadening. The instrumental and physical broadenings of the samples were measured through FWHM, and by utilizing the corrected physical broadening, the average crystallite size could be conveniently calculated using Scherrer's equation as follows:^21,22^2
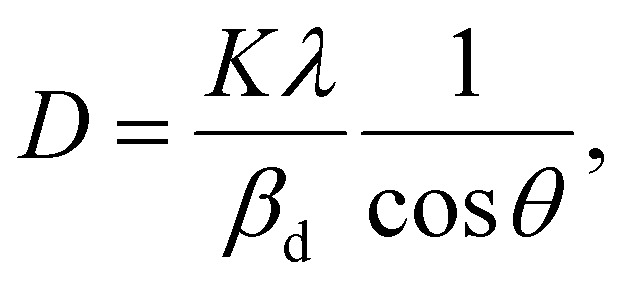
where *D* is the average crystallite size (nm); *K* is the shape factor, usually taken as 0.9 for ceramic materials; *λ* is the wavelength of X-rays; and *θ* is the Bragg angle.

In most studies, the average crystalline size is determined using only for the sharpest peak, so to reduce the sum of absolute error, 
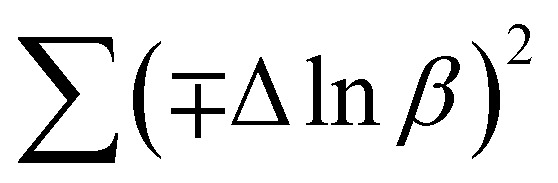
, Monshi *et al.*^23^ suggested the modified Scherrer formula to provide a more accurate value of *D* from all or some of the different peaks.3
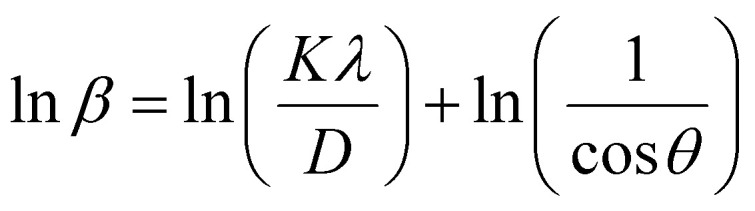


From eqn (3), if the line of ln *β* is drawn in terms of 
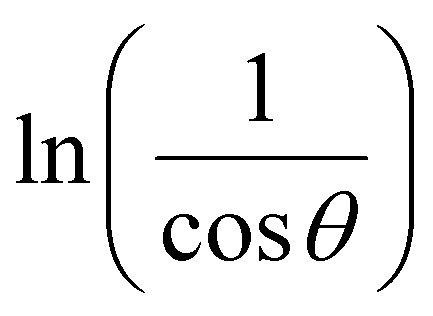
, then the *y*-intercept could give the value of 
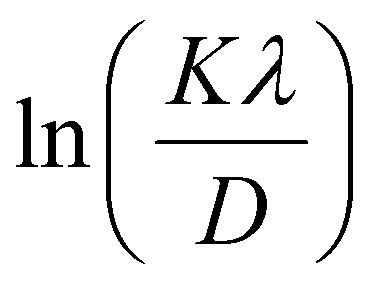
. Thus, the crystal size is as follows:4
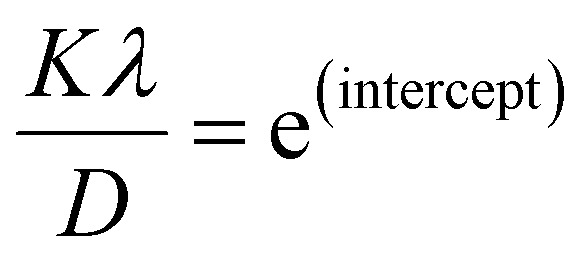


**Fig. 2:**
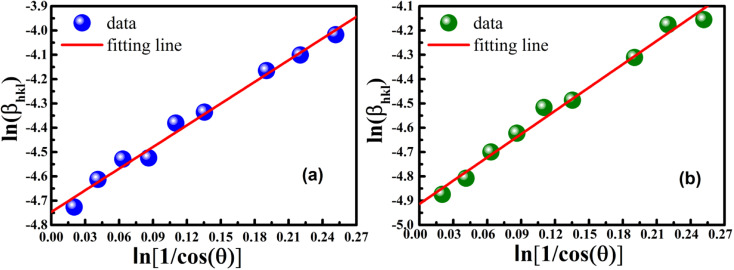
Linear plots of modified Scherrer equation and gained intercepts for different LSMO obtained from (a) S1 and (b) S2.

In order to obtain the more accurate value of crystalline for samples, ln *β vs.*
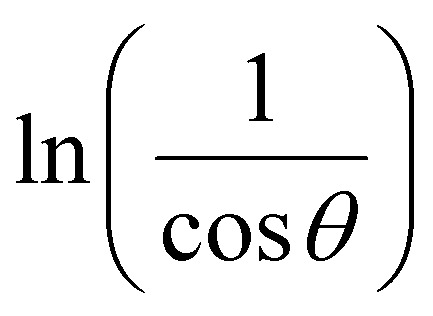
 graph has been plotted for all the diffraction peaks. Fig. 2 displays the graph of ln *β vs.*
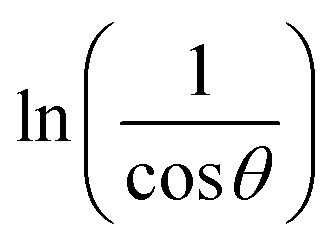
 for both samples of LSMO nanoparticles. The fitting lines determined from eqn (3) for S1 and S2 were *y* = −4.7987 + 2.9748*x* and *y* = −4.9937 + 3.1941*x*, respectively. Therefore, the average crystalline size calculated using eqn (4) was 16.83 nm and 20.45 nm for S1 and S2, respectively.

The modified Scherrer method is the only method for determining the crystalline sizes in this study that could provide checkpoints to evaluate the accuracy of the obtained results. The slope in eqn (5) should be equal to the theoretical slope, *i.e.*, 
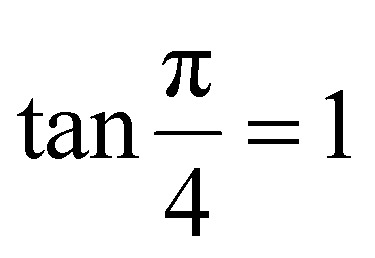
.^23^ However, due to errors associated with the experimental data, the least squares method gives the best slope value and equal to 
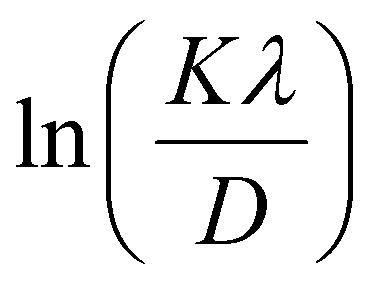
, indicating that some experimental points have to be removed to reduce the error and obtain a more accurate crystalline size. However, for comparison between methods, all the experimental points obtained from the XRD peaks of the samples remained the same.

### 3.2. The Williamson–Hall analysis

Scherrer method only considers the effect of crystal size on the broadening of the XRD peaks; therefore, it does not give any information regarding the internal strain of the lattice, which is developed from the point defect, grain boundary, triple junction, and stacking faults in nanocrystals.^24,25^ Many methods, such as the W–H and Warren–Averbach methods, are utilized to evaluate the effect of the intrinsic strain on the broadening of XRD peaks. Therefore, they could be used to determine the intrinsic strain along the crystal size. Among these methods, the W–H method is an easy, simple, and useful one.^26^ According to this method, the physical expansion of the XRD peaks occurs due to the influence of the size and the microscopic strain of the nanocrystal. Thus, the total broadening could be calculated as follows:

5 *β*_total_ = *β*_size_ + *β*_strain_

In this study, the W–H method included the uniform deformation model (UDM), uniform stress deformation model (USDM), and uniform deformation energy density model (UDEDM) were used and discussed in the following sections.

#### 3.2.1 Uniform deformation model (UDM)

UDM assumes that the strain is uniform in all the crystallographic directions, despite the fact that most of the nanocrystals have imperfect structures. In other words, UDM considers the strain to be isotropic in nature,^21^ and this intrinsic strain affects the physical broadening of the XRD profile. Therefore, the strain-induced broadening (*β*_strain_) could be calculated as follows:

6 *β*_strain_ = 4*ε* × tan *θ*

The *β*_size_ in eqn (5) is given by the Scherrer's expression as follows: 
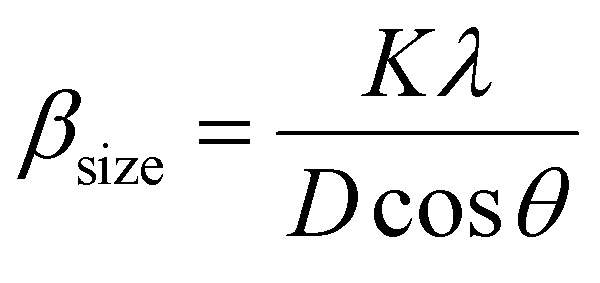
. Therefore, the total resulting broadening due to the strain and size of any diffraction line is7
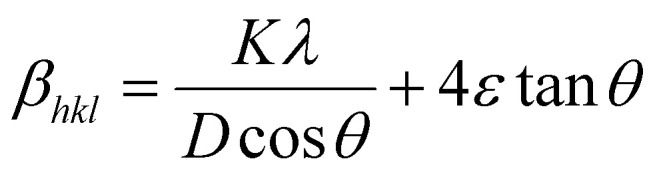
where *ε* is the microstrain. By multiplying both sides of eqn (7) by cos *θ* and when sin *θ* = tan *θ* × cos *θ*, the following equation could be obtained:8
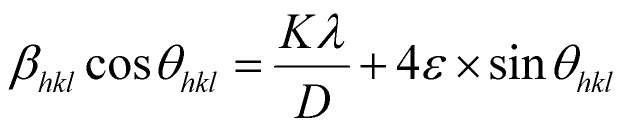


**Fig. 3:**
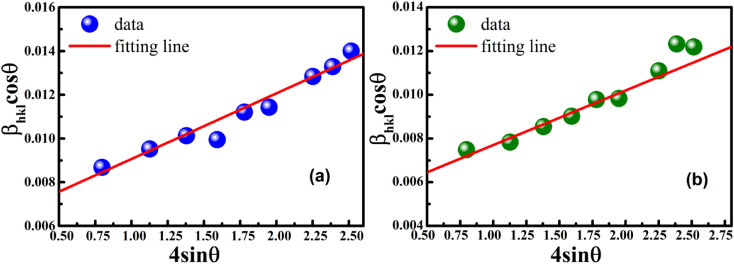
Linear plots of uniform deformation model obtained from (a) S1 and (b) S2.

Eqn (8) shows that if the graph is plotted with *β*_*hkl*_ cos *θ* as the *y*-axis and 4 sin *θ* as the *x*-axis, then the relationship between these two quantities is linear and the line has a slope, directly giving the value of the microstrain (*ε*). Meanwhile, the *y*-intercept is equal to the value of 
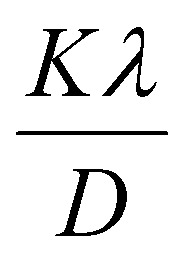
, from which the value of the crystal size could be determined. Fig. 3 shows a plot of *β*_*hkl*_ cos *θ versus* 4 sin *θ*, and the red line denotes a good fitted line. The average crystal sizes determined from UDM were approximately 22.73 and 26.66 nm for S1 and S2, respectively. From the slope, the intrinsic average strain values were calculated to be 3.00 × 10^3^ and 2.49 × 10^3^ for S1 and S2, respectively. A positive value of the intrinsic strain indicates that this strain is a tensile strain, whereas a negative value indicates compressive strain.^24^

#### 3.2.2 Uniform stress deformation model (USDM)

The uniform strain model is based on the assumption that nanocrystals are homogeneous and isotropic in nature, which is not completely true for real crystals. Therefore, to better respond to realistic conditions, the W–H equation must be adjusted so that the lattice deformation strain is anisotropic. This modified model is a USDM, in which the lattice deformation stress is assumed to be uniform along all lattice plane directions and considers a small microstrain present in the particles.

Hooke's law relating to strain shows a linear relationship between stress and strain by the expression *σ* = *εY*_*hkl*_, deducing *ε* = *σ*/*Y*_*hkl*_, where *σ* is the stress of the crystal; *ε* is the anisotropic microstrain, depending on the crystallographic directions; and *Y*_*hkl*_ is the modulus of elasticity or Young's modulus. Therefore, by substituting the value of *ε* in eqn (8), the W–H equation could be modified as follows:^21^9
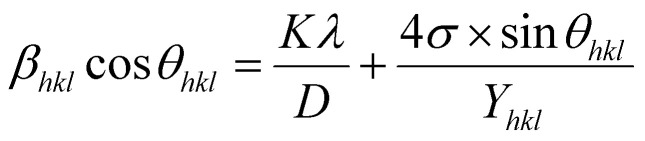


For hexagonal crystals, the Young's modulus is given by the following expression:^25,26^10
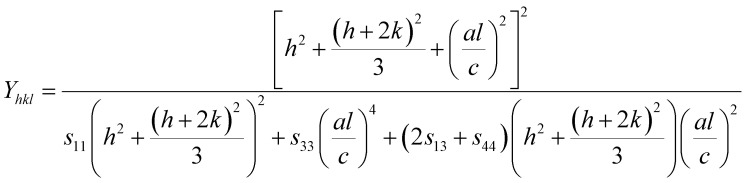
where *h*, *k*, and *l* are the Miller indices, and *a* and *c* are the lattice constants (these values could be extracted from Rietveld refinement analysis using the X'pert software). In addition, *s*_11_, *s*_13_, *s*_33_, and *s*_44_ refer to the elastic compliance values of hexagonal manganites, and they could be determined from the elastic stiffness constants *c*_11_, *c*_12_, *c*_33_, and *c*_44_ by using the following expression:^27^11a
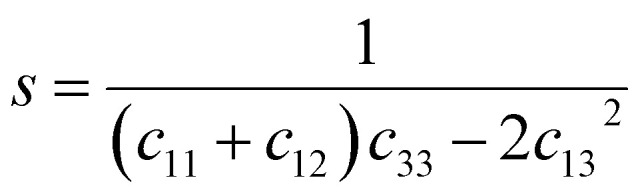
11b
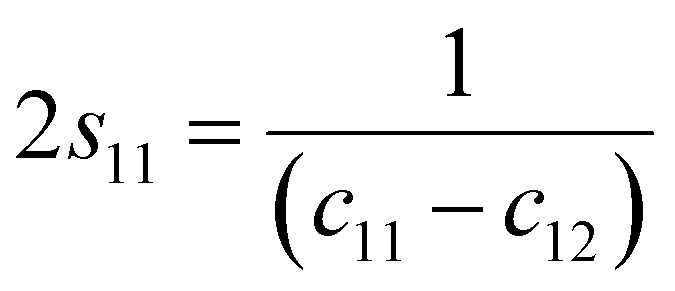

11c *s*_12_ = *c*_33_*s* − *s*_11_

11d *s*_13_ = −*c*_13_*s*

11e *s*_33_ = *s*(*c*_11_ + *c*_12_)

11f
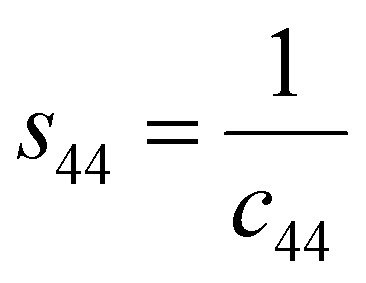


The values of *c*_11_, *c*_12_, *c*_33_ and *c*_44_ for hexagonal manganites are cited in ref. 28. It is to be noted that the value of elastic stiffness constants are common for all the hexagonal manganites. By using these values and those from eqn (11a)–(11f), the elastic coefficients of S1 and S2 were calculated, as shown in Table 1. Furthermore, the Young's modulus (*Y*_*hkl*_) corresponding to the Miller indices of S1 and S2 were calculated using eqn (10), as presented in Table 2. The average *Y*_*hkl*_ values for S1 and S2 were 268.358 and 268.364 GPa, respectively.

**Table 1:** Elastic compliances and stiffness constants of S1 and S2 samples

Elastic compliances (GPa)^28^					Stiffness constants × 10^−3^ (GPa^−1^)				
*c*_11_	*c*_12_	*c*_13_	*c*_33_	*c*_44_	*s*_11_	*s*_12_	*s*_13_	*s*_33_	*s*_44_
248	129	106	308	119	4.20	−0.91	−1.13	4.03	8.40

**Table 2:** Young's modulus (*Y*_*hkl*_) corresponding to the Miller indices of the samples S1 and S2, respectively

S1				S2			
*h*	*k*	*l*	*Y*_*hkl*_ (GPa)	*h*	*k*	*l*	*Y*_*hkl*_ (GPa)
0	1	2	271.965	0	1	2	271.977
1	0	4	276.111	1	0	4	276.103
2	0	2	251.638	1	1	3	251.648
0	2	4	271.965	0	2	4	271.977
1	1	6	278.109	1	1	6	278.105
2	1	4	263.215	2	1	4	263.228
2	0	8	276.111	2	2	0	276.103
0	3	6	271.965	0	3	6	271.977
1	3	4	254.144	1	3	4	254.155

Theoretical studies have shown that *Y*_*hkl*_ is closely related to the cohesive force between atoms. Therefore, the variation of *Y*_*hkl*_ fairly accurately reflects the information of the lattice variation. Compared with the value of 167 GPa of La_0.7_Sr_0.3_MnO_3_ bulks,^29^ the high value of *Y*_*hkl*_ in the samples of the present study showed that the change in JT-type distortion of the MnO_6_ octahedron is very large. Normally, the *R*3̄*C* space group enforces on the average equal Mn–O bond lengths and thus prevents static coherent JT-type distortions of the MnO_6_ octahedron. Although neutron diffraction experiments have shown no obvious evidence for the existence of JT-type distortions in hexagonal or rhombohedral La_0.7_Sr_0.3_MnO_3_,^30^ Louca *et al.*^31^ proposed that the local atomic structure could deviate significantly from the average, and thus, the local JT distortion could persist even if the crystallographic structure has no JT distortion. Therefore, in the case of nano La_0.7_Sr_0.3_MnO_3_, the high value of *Y*_*hkl*_ indicates that the dynamic incoherent local JT distortion of the lattice is significant in the nanoscale samples and fabricated by reactive milling method.

**Fig. 4:**
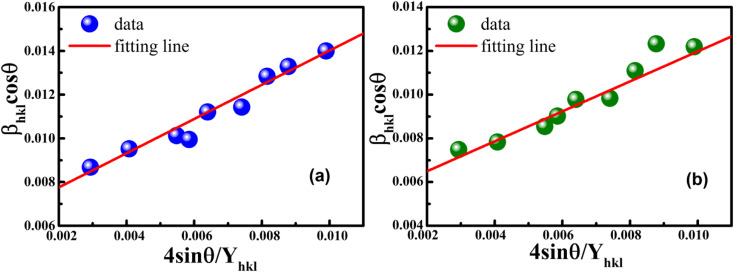
Uniform stress deformation model plots for (a) S1 and (b) S2.

The uniform strain stress (*σ*) from the slope and the average crystal sizes from the *y*-intercept of the linear fit of the plotting of *β*_*hkl*_ cos *θ vs.* 4 sin *θ*/*Y*_*hkl*_ could be estimated. Fig. 4 displays the plotting of *β*_*hkl*_ cos *θ* as a function of 4 sin *θ*/*Y*_*hkl*_, and the red line indicates a good fitted line from eqn (9). The values of *y*-intercept found from the linear fit for S1 and S2 were 0.0062 and 0.0051, respectively, and the average crystal sizes were determined as 22.36 and 27.03 nm, respectively.

In addition, from the slope of linear fitting lines for samples S1 and S2, the values of uniform strain stress (*σ*) were obtained as 0.782 and 0.685 GPa, respectively, and the strain values (*ε*) were calculated as 2.91 × 10^−3^ and 2.55 × 10^−3^, respectively.

#### 3.2.3 Uniform deformation energy density model (UDEDM)

In real crystals, various defects, dislocations, and agglomerations produce imperfections of most crystals. So, the hypothesized isotropic nature of the crystal of UDM and the linear relationship between stress and strain of USDM could not be satisfied completely in the real system. Therefore, UDEDM, which assumes that the deformation energy is uniform in all directions of the crystal, was used as an alternative model to investigate the crystal structure parameters of the material.

The Hooke's law for an elastic system shows the relationship between energy density (*u*) and strain by *u* = (*ε*^2^*Y*_*hkl*_)/2. Therefore, eqn (9) could express the maximum as12
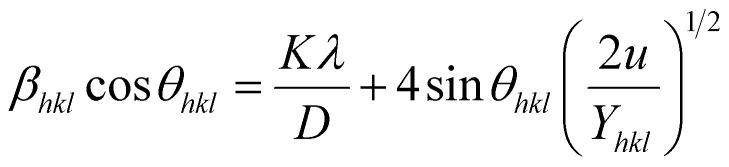


**Fig. 5:**
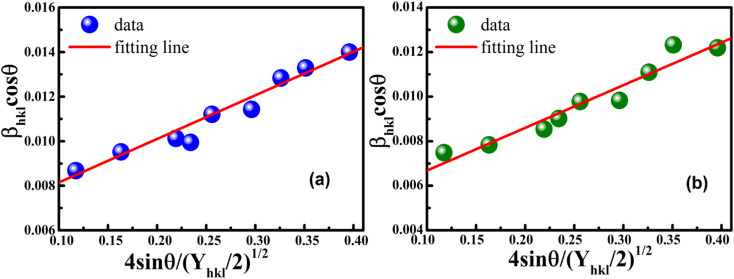
Linear plots of uniform deformation energy density model for (a) S1 and (b) S2.

The anisotropic energy density (*u*) was estimated from the slope of the linear fit and the crystallite size *D* from the *y*-intercept of the plot *β*_*hkl*_ cos *θ*_*hkl*_*vs.* 4 sin *θ*_*hkl*_(2/*Y*_*hkl*_)^1/2^. Fig. 5 shows a plotting of *β*_*hkl*_ cos *θ*_*hkl*_*vs.* 4 sin *θ*_*hkl*_(2/*Y*_*hkl*_)^1/2^, and the red line indicates a linear fit. The values of *u* were determined from the slope of the fitting line to be 0.199 and 0.166 GPa for S1 and S2, respectively. The average crystal sizes determined from the *y*-intercept were 0.0060 and 0.0050 for S1 and S2, respectively, so the crystal size values were 23.10 nm for S1 and 27.51 nm for S2. Given that *σ* = *εY*_*hkl*_ and *u* = (*ε*^2^*Y*_*hkl*_)/2, the stress (*σ*) could be determined by the expression *u* = (*σ*^2^/2*Y*_*hkl*_). The results showed that the strain values (*ε*) were 3.10 × 10^−3^ and 2.38 × 10^−3^ (*Y*_*hkl*_ ∼ average Young's modulus) for S1 and S2, respectively.

### 3.3. Size–strain plot (SSP)

The W–H method assumes that the broadening of peaks is essentially isotropic, thus emphasizing that the diffracting domains are isotropic due to the contribution of the effect of size- and microstrain-induced broadenings, which are considered as Cauchy-like profile. However, in the case of isotropic line broadening, better evaluating the size–strain parameters is possible by considering an average SSP.^25^ The advantage of this method is that less weight is given to the data from reflections at high angles, where accuracy is usually lower. In this approximation, the “crystal size” broadened profile is assumed to be illustrated by the Lorentzian function and the “strain broadened profile” by the Gaussian function. Accordingly,^15,22–24^13
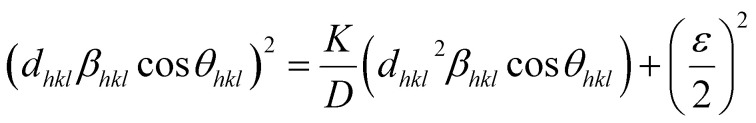
or14
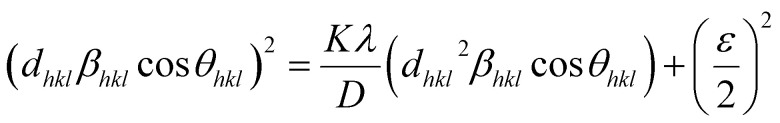


However, the above equations are not appropriate because they do not follow the dimensional homogeneity rule, so the appropriate equation of the SSP method must be as follows:^32,33^15a
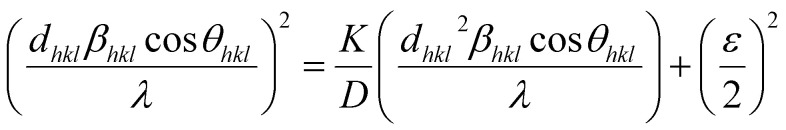
or15b
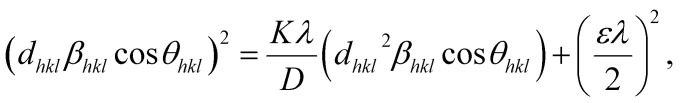
where *d*_*hkl*_ is the lattice distance between the (*hkl*) planes and has a magnitude for the hexagonal crystal as16
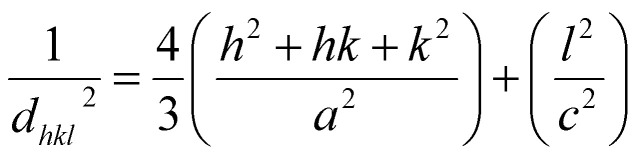


**Fig. 6:**
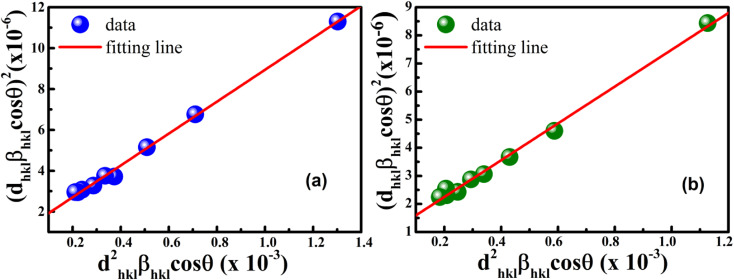
Size–strain plot method of (a) S1 and (b) S2.

According to eqn (15b), the corresponding SSP for samples was obtained by plotting (*d*_*hkl*_*β*_*hkl*_ cos *θ*_*hkl*_)^2^ on *y*-axis with respect to (*d*_*hkl*_^2^*β*_*hkl*_ cos *θ*_*hkl*_) on the *x*-axis for all peaks of S1 and S2 samples with the hexagonal phase. Fig. 6 displays that (*d*_*hkl*_*β*_*hkl*_ cos *θ*_*hkl*_)^2^ is plotted with respect to (*d*_*hkl*_^2^*β*_*hkl*_ cos *θ*_*hkl*_), and the red line indicates a linear fit. The particle size (*D*) values were determined from the slope of the linearly fitted data as 18.01 and 20.83 nm for S1 and S2, respectively, whereas the root of the *y*-intercept provided the strain (*ε*) values of 3.40 × 10^−3^ and 2.76 × 10^−3^ for S1 and S2, respectively.

### 3.4. Halder–Wagner (H–W) method

The SSP method assumes that the crystalline size broadening of the XRD peak profile is described by the Lorentzian function, whereas the strain broadening corresponds to the Gaussian function. However, the XRD peaks actually are neither Lorentzian nor Gaussian functions, so the Halder–Wagner method is a useful alternative method to determine the crystalline size and strain of sample. This method is based on the assumption that the broadening of the XRD peaks is a Voigt symmetric function,^34^ and that it could be analyzed as deconvolution of two Lorentzian and Gaussian functions. In this case, the FWHM of the physical profile is written as follows:

17 *β*_*hkl*_^2^ = *β*_L_*β*_*hkl*_ + *β*_G_^2^,

where *β*_L_ and *β*_G_ are the FWHM of the Lorentzian and Gaussian functions, respectively. The H–W method has the advantage that it offers more weight to the peaks at low and medium diffraction angles and minimizes the overlap of the diffraction peaks.^32^ According to the Halder–Wagner method, the relation between the crystallite size and lattice strain could be expressed as follows:18
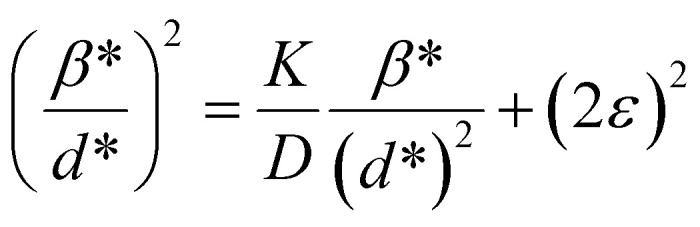
where β* = *β*_*hkl*_ cos *θ*/*λ* and d* = 2 sin *θ*/*λ*. So, eqn (18) could be modified as19
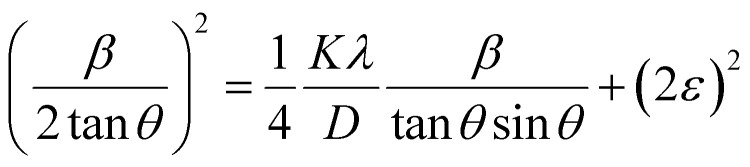


**Fig. 7:**
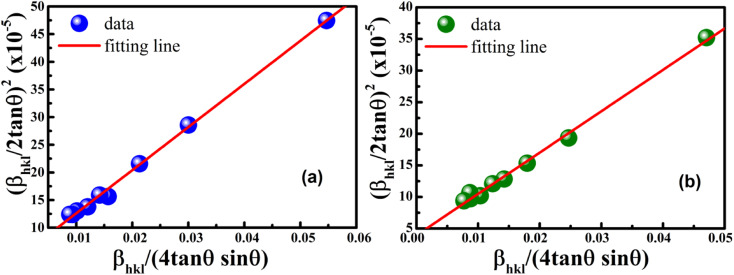
Linear plots of Halder–Wagner method for (a) S1 and (b) S2.

To estimate the values of crystallite size and strain using eqn (19), we fitted a linear equation to the 
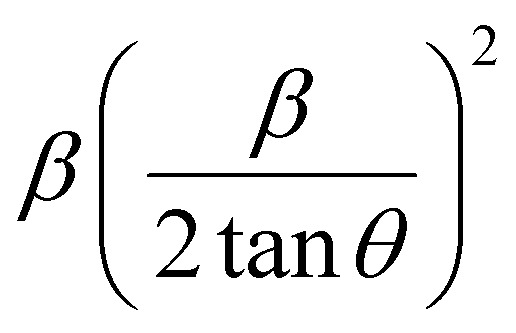
*versus*
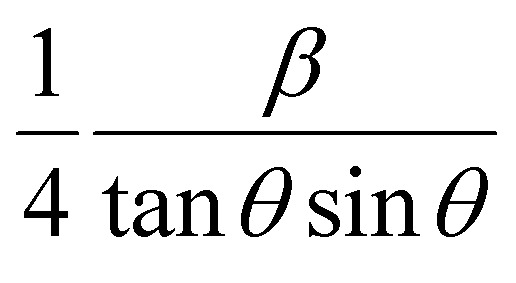
 plots (see Fig. 7). The slope of the plotted straight line provides the average crystalline size, whereas the intercept gives the intrinsic strain of the samples. The average particle sizes were calculated from the plot as 17.78 nm for S1 and 21.20 nm for S2, which matched well with those obtained from the SSP model. Meanwhile, the calculated strain values from the Halder–Wagner plot were 3.46 × 10^−3^ for S1 and 3.12 × 10^−3^ for S2.

Table 3 provides all the calculated values of the average crystalline size and the intrinsic strain, including other elastic parameters. The strain (*ε*) values calculated using the methods were comparable and consistent with one another. Furthermore, the average crystalline size (*D*) values obtained by Modified-Scherrer, SSP and H–W methods are almost similar, whereas these values are slightly higher for W–H method. Such difference could be attributed to both methods considering the anisotropy nature of the elastic constant are primarily different. USDM shows that deformation stress (*σ*) is the same in all crystallographic directions, with energy density (*u*) being anisotropic, whereas UDEDM assumes that deformation energy is uniform in all crystallographic directions, so deformation stress (*σ*) is anisotropic. According to literature,^19,21,24^ the SSP and H–W methods are more suitable than W–H, because the data from reflections at high angles are less important, and the data points are located very close to linear fit.

**Table 3:** Geometric parameters of La_0.7_Sr_0.3_MnO_3_ nanoparticles calcinated at 700 °C (S1) and 800 °C (S2) for 4 h

Sample	Modified Scherrer method	Williamson–Hall method									Size–strain plot		Halder–Wagner method	
		UDM		USDM			UDEDM							
	Size *D* (nm)	Size *D* (nm)	Strain *ε* (10^−3^)	Size *D* (nm)	Strain *ε* (10^−3^)	Stress *σ* (GPa)	Size *D* (nm)	Strain *ε* (10^−3^)	Stress *σ* (GPa)	Energy density *u* (GPa)	Size *D* (nm)	Strain *ε* (10^−3^)	Size *D* (nm)	Strain *ε* (10^−3^)
S1	16.83	22.73	3.00	22.36	2.91	0.782	23.10	3.10	0.831	0.199	18.01	3.14	17.78	3.46
S2	20.45	26.66	2.49	27.03	2.55	0.685	27.51	2.38	0.639	0.166	20.83	2.76	21.20	3.12

### 3.5. Morphological study

**Fig. 8:**
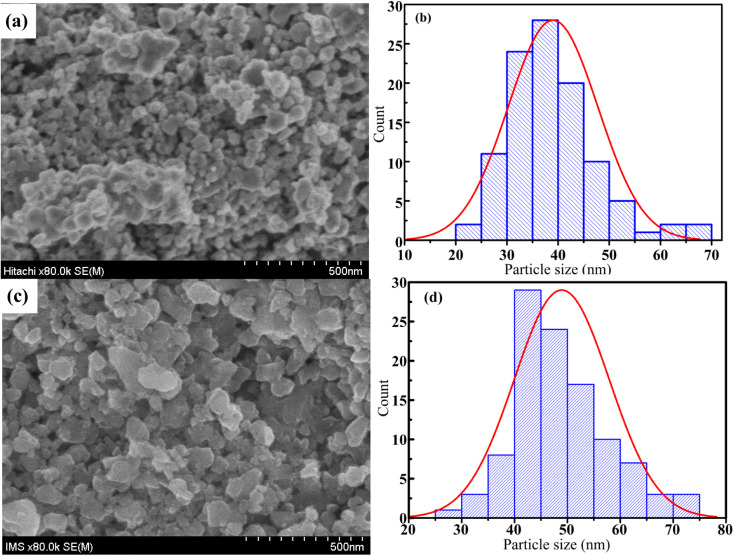
FESEM micrograph (a and c) and grain size distribution histograms (b and d) of S1 and S2 samples.

FESEM images are one the best way to study of the morphology of the La_1−*x*_Sr_*x*_MnO_3_ (*x* = 0.3) nanoparticles. The FESEM images and the particle-size distribution for the S1 and S2 nanoparticles are presented in Fig. 8. All the samples consisted of agglomerated or interconnected nanoparticles with non-uniform morphology may be due to the magnetic dipole–dipole interactions, which caused the nanoparticles to aggregate.^35^ The particle sizes were in the range of 20–70 nm for S1 and 25–75 nm for S2. The average particle size was determined by ImageJ software, and the Gaussian function showed that the average particle sizes of S1 and S2 were ∼39 and ∼50 nm, respectively, as shown in Fig. 8(b) and (d).

## 4. Conclusions

In this study, nano-polycrystalline La_1−*x*_Sr_*x*_MnO_3_ (*x* = 0.3) samples were synthesized using the reactive milling technique with conventional sintering method. The room-temperature X-ray diffraction analysis showed that all the studied compounds possessed a hexagonal structure with a *R*3̄*C* space group. The average crystalline size and the various elastic properties of the La_1−*x*_Sr_*x*_MnO_3_ (*x* = 0.3) nanomanganites, such as intrinsic strain, stress, and energy density, were determined by different methods, such as the modified Scherrer, W–H plot, SSP, and Halder–Wagner methods, *via* XRD peak broadening analysis. The calculation results showed that the SSP and Halder–Wagner methods are the two most suitable methods to accurately determine the crystal size and intrinsic strain of nanomanganites. Finally, the morphology and the distribution of the average particle size were studied *via* FESEM, which showed that the average size increased from 39 nm for S1 to 49 nm for S2 as the annealing temperature increased from 700 °C to 800 °C.

## Author contributions

Do Hung Manh: conceptualization, investigation, formal analysis, data curation, writing. Tran Thi Ngoc Nha, Le Thi Hong Phong and Pham Hong Nam: investigation, formal analysis. Tran Dang Thanh and Pham Thanh Phong: conceptualization, investigation, formal analysis, data curation, supervision, project administration, writing – review & editing.

## Conflicts of interest

The authors declare that they have no conflict of interest.

## Supplementary Material
